# Critically raw materials as potential emerging environmental contaminants, their distribution patterns, risks and behaviour in floodplain soils contaminated by heavy metals

**DOI:** 10.1038/s41598-023-36477-7

**Published:** 2023-06-13

**Authors:** Radoslava Kanianska, Marek Drimal, Jozef Varga, Michael Komárek, Samuel Kudjo Ahado, Milada Šťastná, Miriam Kizeková, Ľubica Jančová

**Affiliations:** 1 Faculty of Natural Sciences, Matej Bel University Banská Bystrica, Tajovského 40, 974 01 Banská Bystrica, Slovakia; 2 Faculty of Environmental Sciences, Czech University of Life Sciences Prague, Kamýcká 129, 165 00 Praha-Suchdol, Czech Republic; 3 Faculty of Agrobiology, Food and Natrual Resources, Czech University of Life Sciences Prague, Kamýcká 129, 165 00 Praha-Suchdol, Czech Republic; 4 Department of Applied and Landscape Ecology, Faculty of AgriSciences, Mendel University in Brno, Zemědelská 1, 613 00 Brno, Czech Republic; 5 National Agricultural and Food Centre, Research Institute of Plant Production,Grassland and Mountain Agriculture Institute, Mládežnícka 36, 974 21 Banská Bystrica, Slovakia

**Keywords:** Environmental chemistry, Environmental impact

## Abstract

The expanding demand for new critical raw materials can lead to their increased release to the environment in the form of emerging environmental contaminants (EECs). However, there has never been a comprehensive study that takes into account the total EEC content, the content of various EEC fractions, their behaviour in floodplain soils, and potential ecological and human health risks. The occurrence, fractions, and influencing factors of the seven EECs (Li, Be, Sr, Ba, V, B, Se) originating from historical mining in floodplain soils of various ecosystems (arable lands, grasslands, riparian zones, contaminated sites) were investigated. Based on the evaluation of the overall levels of EECs (potentially toxic elements) in comparison to the soil guideline values set by European legislation for Be, Ba, V, B, and Se, it was found that only Be did not exceed the recommended limits. Among the elements analyzed, Li had the highest average contamination factor (CF) of 5.8, followed by Ba with 1.5 and B with 1.4. Particularly concerning was the discovery of a potential serious health risk associated with Li exposure for children, as indicated by hazard quotients ranging from 0.128 to 1.478. With the exception of Be and Se, the partitioning of the EECs into the different fractions revealed that the EECs are primarily bound with the residual fraction. Be (13.8%) had the highest percentage of exchangeable fraction as the most bioavailable in the first soil layer, followed by Sr (10.9%), Se (10.2%), Ba (10.0%), and B (2.9%). The most frequently observed correlations were between EEC fractions and pH/KCl, followed by soil organic carbon and manganese hydrous oxides. Variance analyses confirmed the impact of different ecosystems on EEC total content and fractions.

## Introduction

Globally, the demand for new critical raw materials (CRMs) is growing very quickly. The 2020 EU list contains 30 materials including lithium, beryllium, strontium, vanadium, baryte (barium sulphate) and borates (naturally occurring minerals containing boron)^1^. Selenium was identified as a critical material in the 2011 assessment^2^. This growing global importance and demand for CRMs can lead to undesirable increased in their release to the environment. Therefore, many of these CRMs have also attracted attention from the scientific community as emerging environmental contaminants (EECs). Environmental contamination of these elements can be related to their extensive use, e.g. Li in mobile phones and mood-stabilizing drugs^3^; Be in electronics, telecommunication, nuclear power and industry^4^; barium compounds are used as fillers or additives in industrial products; vanadium is used as an additive in steel and titanium alloys and as a catalyst for chemicals; borates are important ingredients in a variety of household and commercial products. To date, research has not succeeded to provide a clear picture of how increased production of CRMs as EECs adversely affects all compartments of the environment^3^. There is a wide variety of inorganic and organic EECs^5^.

The release of industrial inorganic EECs into the environment can also be related to mining activities that are mainly responsible for the release of traditional environmental contaminants (TECs). EECs that were not recognized before or attracted less attention do not exert acute toxicity but exert their effects in more hidden ways^6^. Additionally, increased use and disposal of CRMs can contribute to increased exposure of EECs. Drinking water and plants often provide the primary pathway of exposure for many EECs to the food chain^7^. Both depend on the behaviour of EECs in soil which can be entirely different from TECs^6^. There is a lack of information on the distribution patterns and behaviour of many EECs in the soil. The areas suitable for this research are those in which past mining activities have left negative environmental footprints related to soil contamination by heavy metals. Heavy metal accessories can simply be EECs with different chemical properties. In this regard, special attention should be paid to floodplain soils. In floodplains, contamination can occur from both geogenic and anthropogenic sources. Additionally, drainage systems and water flows can contribute to the transport and spread of contamination. Furthermore, contamination can negatively affect even distant rare natural and artificial alluvial ecosystems with a possible negative impact on human health.

The purpose of this research is to look into the distribution patterns and behavior of selected EECs (Li, Be, Sr, Ba, Ti, V, B, and Se) in the Štiavnica River (SR) floodplain soils that have been contaminated by heavy metals from historical mining. In this study, we aimed at: (1) to specify the total content and the different fractions of EECs in soils; (2) to calculate soil contamination indices; (3) to assess human health risk; (4) to evaluate the distribution patterns of EECs; (5) to find the relationships between soil characteristics and the EEC fractions; (6) to assess the impact of different factors on the behaviour of EECs in soil. Our study is novel because selected EECs have not yet been studied in such an intensive and thorough way in floodplain soils, taking into account the total content, the various fractions, their relationships in floodplain soils, and potential ecological and human health risks.

## Material and methods

### Study area

Sampling sites were selected in the floodplain along the SR in central Slovakia. The riverbanks consist of fluvial deposits (sand and silt). The SR is 55 km long, with a 443 km^2^ large catchment area. The Štiavnické vrchy Mountains, Podunajská pahorkatina Hills, and Krupinská planina Plain are the main physiographic divisions that characterize this drainage basin. In the upstream part of the basin in the Štiavnické vrchy Mounatins of volcanogenic origin, the relief is dominated by hills and valleys with forests and partly by permanent grasslands used as meadows. The Štiavnické vrchy Mountains are a type of neovulcanic mountain range. They consist of pyroclastic rocks, primarily andesite tuff agglomerates, andesites and rhyolites. The region hosts rich metal, silver and gold mineralizations that have been the basis for a long lasting mining tradition. In the eighteenth century, the region became the biggest mining centre in the Habsburg Monarchy^8^. In the downstream part of the basin in the Podunajská pahorkatina Hills, the relief is dominated by a flat surface. The bedrock consists of clay- and sand-stones on slopes, sand and gravel in areas of floodplains. The land is used as arable land.

### Sampling and field measurements

**Figure 1:**
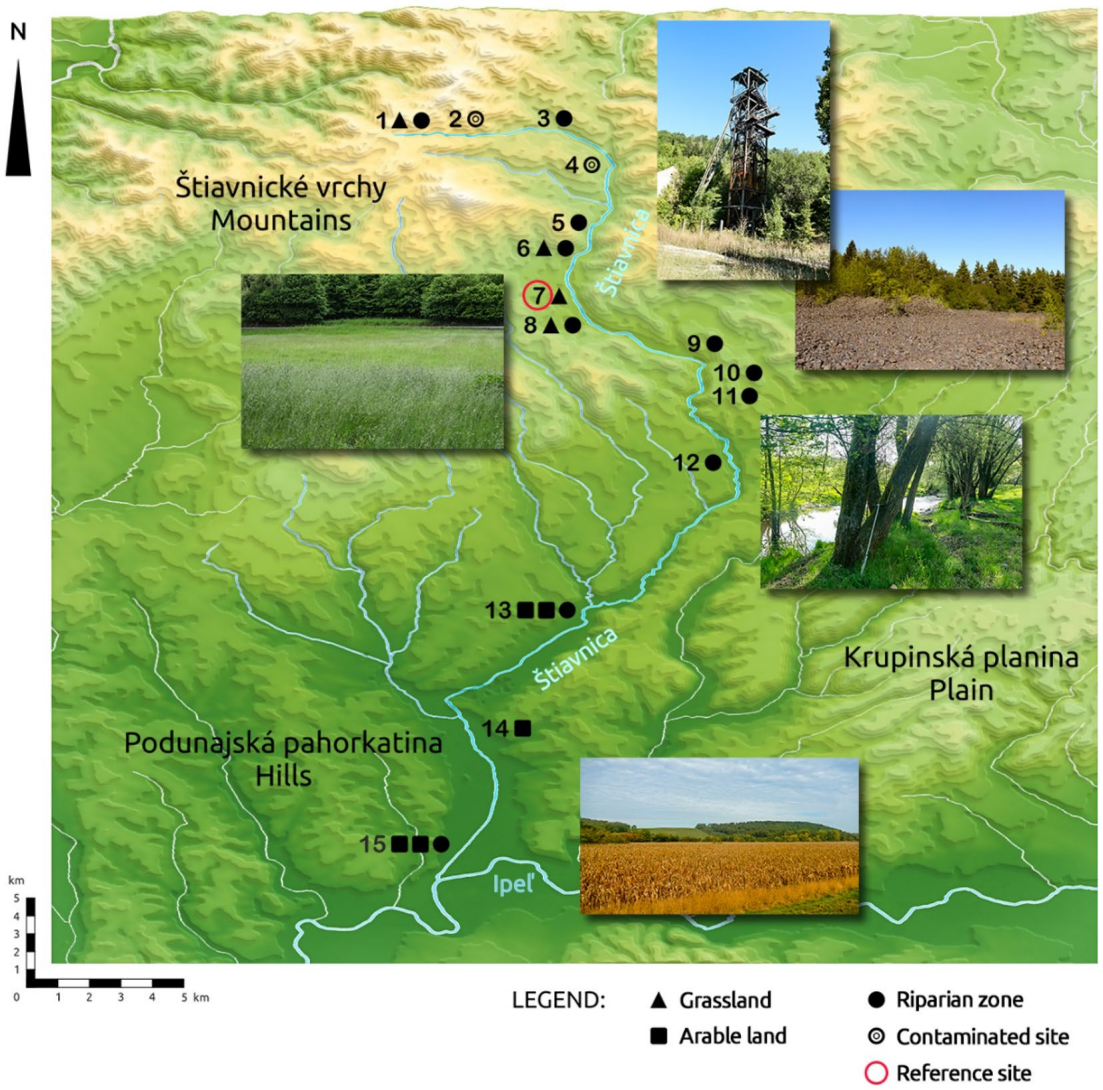
Isometric map of the sampling points and photos from selected study sites (photos R. Kanianska). The map was generated using 3D Map Generator, ver. 1.5, https://www.3d-map-generator.com/3d-map-generator-terrain/.

We collected a total of 42 soil samples from two layers (0–10 and 20–30 cm) from two contaminated sites (CS, classified as Ekranic Technosols), 1 reference site (RS, classified as Haplic Cambisol), and 12 alluvial sites (AS, classified as Haplic Fluvisols). The 12 AS were located along the continuous elevation gradient of the SR, from the highest elevation (659 m a.s.l.) down to an elevation of 123 m a.s.l. (Fig. 1, Table 1).

**Table 1:** Site characteristics.

Study site name	Latitude	Longitude	Altitude (m)
2CS–PEB	48° 26′ 35.2″	18° 53′ 05.7″	659
4CS–CEB	48° 25′ 56.1″	18° 55′ 37.0″	487
1AS–RZ	48° 26′ 06.9″	18° 52′ 38.8″	648
1AS–GL	48° 26′ 07.9″	18° 52′ 38.8″	648
3AS–RZ	48° 26′ 18.3″	18° 54′ 12.4″	518
5AS–RZ	48° 23′ 39.6″	18° 55′ 66.9″	376
6AS–RZ	48° 23′ 00.6″	18° 55′ 52.8″	351
6AS–GL	48° 23′ 00.6″	18° 55′ 51.5″	353
8AS–RZ	48° 20′ 51.4″	18° 55′ 55.3″	284
8AS–GL	48° 20′ 49.6″	18° 55′ 53.2″	288
9AS–RZ	48° 19′ 55.1″	18° 57′ 58.3″	260
10AS–RZ	48° 19′ 38.5″	18° 58′ 43.3″	251
11AS–RZ	48° 18′ 99.0″	18° 58′ 42.9″	239
12AS–RZ	48° 17′ 42.0″	18° 58′ 55.9″	217
13AS–RZ	48° 12′ 18.0″	18° 55′ 37.3″	148
13AS–AL1	48° 12′ 15.0″	18° 55′ 40.9″	150
13AS–AL2	48° 12′ 17.1″	18° 55′ 25.8″	150
14AS–AL	48° 09′ 26.6″	18° 52′ 45.0″	137
15AS–RZ	48° 05′ 09.6″	18° 52′ 20.9″	123
15AS–AL1	48° 05′ 11.2″	18° 52′ 19.6″	123
15AS–AL2	48° 05′ 24.3″	18° 52′ 08.7″	123
7RS–GL	48° 21′ 41.7″	18° 55′ 12.8″	335

*CS* contaminated site, *PEB* probable environmental burden, *CEB* confirmed environmental burden, *AS* alluvial site, *RS* reference site, *RZ* riparian zone, *GL* grassland, *AL* arable land.

The sampling points were located on the river bank within the riparian zones (RZ) in the Štiavnické vrchy Mountains, and on arable land (AL) in the Podunajská pahorkatina Hills. In the case of a larger floodplain, we also collected samples distant from the water flow at the end of the floodplain, that were used as permanent grasslands (GL) in the Štiavnické vrchy Mountains, and as arable land in the Podunajská pahorkatina Hills (at 5 of 12 alluvial sites). Outside the river floodplain, we selected the two contaminated sites (CS), which are poorly developed on metallurgical slag and shallow soils. As CS sites, we selected the area of the Maximilian shaft that is registered in the Register of environmental burdens of the Slovak republic as probable environmental burden (PEB), and the hard rock mine tailing Lintich that is registered as a confirmed environmental burden (CEB), where hazardous substances caused by mining activities can pose a risk to human health or to the environment, soil and groundwater. The Maximilian shaft area was located approximately 1 km and the Lintich mine tailing approximately 100 m from the Štiavnica River.

The RS site represents a site with a ‘normal’ level of contaminants and arising from a combination of natural and diffuse pollution contributions. The RS site was used to determine the background values. The RS site was located about 400 m away from the Štiavnica River outside the floodplain on a slope and is used as a meadow. The soil type was classified as Haplic Cambisol.

Soil samples were collected in the study area in September 2018. Five subsamples were taken with a stainless-steel blade, and then mixed thoroughly to get a composite soil sample. The collected soil samples were stored in a polyethylene bag, transported to the laboratory where they were air-dried at ambient temperature, crushed, and sieved through a 2-mm mesh.

The volumetric soil moisture (SM in %) and salinity (SAL in mS/m) were measured at the same time and places of soil samples collection with WET Sensor (type HH2, Delta-T Devices Ltd., Cambridge, UK), by taking the average of five measurements. Similarly, the redox potential (Eh) was measured by the Oxygen Diffusion meter (Ejkelkamp).

### Analytical methods

The soil chemical parameters were measured in November and December 2018. We determined soil chemical properties such as pH in H_2_O (with a ratio of 1:2.5) and 1 M KCl solution (with a ratio of 1:2.5), total soil organic carbon (SOC) using the oxidimetric method according to Tyurin that is similar to the Walkley–Black oxidation method. The Tyurin titrimetric method is a wet combustion method, where soil organic matter is oxidized by 0.2 M K_2_Cr_2_O_7_ with H_2_SO_4_. After oxidation, excess dichromate is determined by titration with a Mohr´s salt solution. The content of humic and fulvic acids was determined by the short fractionation method according to Kononova and Belchikova using extraction with 0.1 M Na_4_P_2_O_7_·10H_2_O. Oxalate-extractable Al, Fe, and Mn as an indicator of hydrous oxides, were determined by the acid oxalate extraction method as described by Pansu and Gautheyrou with a (NH_4_)_2_-oxalate solution adjusted to pH 3, shake for 4 h in the dark, and centrifuge for 10 min at 10,000*g*. Particle size analysis was performed by pipette method using soil particle sedimentation and particle size fractions (sand, silt, clay) were classified according to the United States Department of Agriculture (USDA) system.

We investigated the total content and content in four chemical forms of seven EECs from various periodic table groups (Li, an alkali metal; Be, Sr, Ba, alkaline earth metals; V, a transition metal; B, metalloid; Se, a nonmetal). Total dissolution of soil samples was performed using a microwave unit (Anton-Paar, Austria). The dissolution method used was an EPA 3015A^9^: 9 mL of HNO_3_, 3 mL of HCl and 1 mL of HF were added to a 0.250-g dry soil sample.

The Community Bureau of Reference Sequential Extraction Procedure (BCR SEP) as described by Rauret et al.^10^ was used. The exchangeable phase (I) was leached with HOAc, the reducible fraction (II) bound to Fe and Mn oxides was leached with NH_2_OH-HCl, the oxidisable fraction (III) bound to organic matter and sulphides was leached with H_2_O_2_ in NH_4_OAc, and the residual fraction (IV) bound to silicate minerals was calculated as the difference between the total content of EECs and the sum of the three fractions. The procedure followed was inductive coupled plasma optical emission spectrometry (ICP-OES).

### Soil pollution assessment

We evaluated soil contamination by EECs using the contamination factor (CF) according to Varol^11^ and the pollution load index (PLI) according to Tomlinson et al.^12^. The CF was calculated according to the formula:

$$\mathrm{CF}=\frac{\mathrm{Ci }}{\mathrm{Cb}},$$

where Ci is the measured content of EEC in soil and Cb is the background value of the EEC content of the reference site. According to Varol^11^, CF < 1 means low contamination, 1–3 is moderate contamination, 3–6 is considerable contamination, and CF > 6 means very high contamination.

The PLI was calculated according to the formula:

$$\mathrm{PLI}=\sqrt[\mathrm{n}]{\mathrm{CF}1\times \mathrm{CF}2\times \mathrm{CF}3\dots \mathrm{CFn}},$$

where n is the number of elements. The PLI > 1 indicates pollution.

### Human health risk assessment

Potential human health risk was also evaluated. In the study, ingestion of soils contaminated with EECs was considered as the main pathways for risk assessment. We calculated the average daily dose (ADD, mg element kg^−1^ bodyweight day^−1^) for children, adult male and adult females:

$$\mathrm{ADD}=\mathrm{C}\times \frac{\mathrm{IR}\times \mathrm{EF}\times \mathrm{ED}{\times 10}^{-6}}{BW\times AT},$$

where C is total soil element content; IR is soil ingestion rate (children: 200; adults: 100 mg dust day^−1^); EF is exposure frequency (children: 350; adults: 250 days year^−1^); ED is exposure duration (children: 6 years; adults: 25 years); BW is bodyweight (children: 15 kg; adults males: 68 kg; adults females: 58 kg); AT is averaging time (children: six 365-day years = 2190 days; adults: 9125 days). Values were obtained as per by Rinklebe et al.^13^, as originally reported in USEPA^14^.

Then hazard quotient (HQ) was calculated to evaluate non-carcinogenic human health risk:

$$\mathrm{HQ}=\frac{\mathrm{ADD}}{RfD},$$

where RfD (in mg element kg^−1^ bodyweight day^−1^) is the EEC oral reference dose: for Be = 0.002, Ba = 0.07, V = 0.007, Se = 0.005^14^, Sr = 0.6^15^, B = 0.2^16^. The RfD for lithium is not available on the Integrated Risk Information System of USEPA. However, the USEPA has developed a provisional peer reviewed toxicity value for lithium with provisional p-RfD of 0.002. The provisional subchronic and chronic RfD for lithium was derived from the lowest observed adverse effect level (LOAEL) from the therapeutically used lithium of 2.1 mg/kg-day for adverse effects in several organs and systems. Dividing the LOAEL of 2.1 mg/kg-day by an uncertainty factor of 1000 yields a subchronic and chronic pRfD of 0.002 mg/kg-day^17^. We think that this value can be used for health risk assessment of soil because exposure through environmental compartments should be minimal to zero.

Then we calculated hazard index (HI) as the sum of HQ values of all EECs. Values of HQ and HI of > 1 indicate high probability of the occurrence of adverse health effects.

### Statistical analysis

The study analyzed the data using the Pearson correlations, principal component analysis (PCA), one-way analysis of variance (ANOVA), and hierarchical cluster analysis (HCA). The SPSS Statistics 28 software was used.

### Soil samples collection

In Slovakia, it is allowed to collect soil samples for research purposes.

## Results

### Soil properties, soil contamination indices and potential health risk assessment indices

The basic soil properties are reported in Table 2.

**Table 2:** Soil properties in the depth of 0–10 and 20–30 cm in contaminated, alluvial and reference sites in different ecosystem types.

Depth cm	Site-Land use		pH/H_2_O	pH/KCl	Eh mV	SOC g/kg	HA/FA	Al_o_ mg/kg	Fe_o_ mg/kg	Mn_o_ mg/kg	Content (%) of			SAL	SM
											Clay	Silt	Sand	mS/m	%
0–10	CS	*x̄*	7.46	7.19	389.5	24.30	0.57	1373.8	11,398.5	4817.9	7.6	29.9	62.6	126.6	12.5
	(n = 2)	SD	0.09	0.21	29.5	9.30	0.02	573.4	4312.0	3708.5	0.3	5.8	6.1	24.1	2.7
	AS-RZ	*x̄*	6.25	5.50	331.6	23.47	0.40	1353.9	7550.1	2833.6	7.4	32.4	60.3	81.8	23.8
	(n = 11)	SD	0.74	0.94	196.7	6.14	0.08	390.3	2280.2	2891.0	2.4	18.0	20.1	40.7	6.4
	AS-GL	*x̄*	5.96	5.47	381.3	21.0	0.42	1278.0	6099.0	3999.2	6.7	37.1	56.3	82.2	26.3
	(n = 3)	SD	0.42	0.12	13.9	1.70	0.03	272.3	3681.2	4125.0	0.3	10.9	10.7	13.6	1.8
	AS-AL	*x̄*	6.53	5.62	379.2	15.09	0.55	1257.8	6802.3	2037.8	18.0	51.5	30.3	109.1	28.1
	(n = 5)	SD	0.50	0.39	10.8	3.76	0.14	407.0	364.6	1014.5	12.3	9.5	15.8	26.1	6.3
	RS-GL (n = 1)	*x*	5.74	4.59	364.0	34.90	0.32	2625.7	2876.4	740.5	25.8	43.6	30.6	101.8	29.7
20–30	AS-RZ	*x̄*	6.11	5.30	327.9	17.05	0.43	1517.7	10,667.8	3352.6	8.3	30.8	60.9	91.4	21.1
	(n = 12)	SD	0.76	0.94	199.2	6.07	0.11	411.4	2655.4	3914.1	3.4	16.7	19.6	52.0	8.6
	AS-GL	*x̄*	5.93	4.96	347.0	12.7	0.44	1293.0	1285.0	4714.3	6.2	31.7	62.1	105.5	18.3
	(n = 3)	SD	0.47	0.45	10.2	2.45	0.04	314.8	2594.8	5470.1	1.5	15.7	16.6	37.4	1.5
	AS-AL	*x̄*	6.76	5.73	351.8	11.69	0.49	1345.6	7217.3	1764.0	19.3	54.8	25.9	159.1	24.3
	(n = 5)	SD	0.41	0.37	29.0	3.55	0.07	505.6	80.3	922.1	10.3	10.5	11.4	24.3	7.0
	RS-GL (n = 1)	*x*	5.77	4.73	324.0	20.10	0.39	2914.4	3518.0	991.9	22.9	43.7	33.4	156.9	34.2

*AS* alluvial site, *CS* contaminated site, *RS* reference site, *AL* arable land, *GL* grassland, *RZ* riparian zone, *Eh* redox potential, *SOC* soil organic carbon, *HA/FA* ratio of humic acid carbon to fulvic acid carbon; _*o*_ extracted by oxalate, *SAL* salinity, *SM* soil moisture.

The total content of EECs in the two soil layers of the SR floodplain together with soil guideline values (SGV) as a range in European legislation^18^ is presented in Table 3. At least one lower SGV limit set up for 5 selected EECs (Be, Ba, V, B, Se) was exceeded in the case of all selected EECs with the exception of Be. Some exceedances were recorded in the CS, AS and RS localities, in both layers, and in all ecosystem types (CS, AL, GL and RZ). For Ba and Se, the upper SGV limit was even exceeded, but only in one locality. The contamination risk in the CS and AS localities was confirmed by the CF and PLI along the whole SR floodplain for 5 EECs (Li, Sr, Ba, V, B) (Table 4). The CF and PLI were not calculated for 2 EECs (Be, Se) due to the appearance of zero values. We measured the lowest content of these two elements in soil with no SGV exceedance in the case of Be and only a few exceedances in the case of Se. In the CS localities, the average CF showed moderate contamination by Li. At AS localities, the average CF showed considerable contamination by Li, moderate contamination by Ba and B. The highest CF was quantified for Li (15.6 in 13AS-AL2), Ba (2.6 in 15AS-AL2) and B (2.3 in 8AS-RZ). In many AS localities, contamination by EECs was similar to or even higher than in CS localities. The PLI indicated that all AS sites were contaminated by EECs (PLI > 1) except one AS locality (3AS-RZ).

**Table 3:** Total content (T) of emerging environmental contaminants in the depth of 0–10 and 20–30 cm in contaminated, alluvial and reference sites with basic statistical characteristics compared to soil guideline values (mg/kg).

Depth (cm)	Site	Locality	Alkali metal	Alkaline earth metals			Transition metal	Metalloids	Non-metal
			Li	Be	Sr	Ba	V	B	Se
0–10	CS	2CS-PEB	20.78	0.98	60.76	24.82	**111.84**	**22.98**	0.00
		4CS-CEB	37.80	1.25	115.57	**108.99**	**134.53**	**28.15**	0.00
		*x̄*	*29.29*	*1.12*	*88.16*	*66.91*	*123.18*	*25.56*	*0.00*
		SD	*8.51*	*0.14*	*27.41*	*42.08*	*11.35*	*2.58*	*0.00*
	AS	1AS-RZ	34.85	1.02	85.46	**368.66**	**133.85**	**24.77**	0.00
		1AS-GL	131.74	0.00	27.94	**227.54**	91.82	**31.94**	**5.99**
		3AS-RZ	19.99	0.76	88.74	**180.55**	93.33	**20.27**	0.00
		5AS-RZ	28.07	0.92	98.37	**408.19**	**131.50**	**29.87**	0.00
		6AS-RZ	22.14	0.94	177.49	**416.20**	**173.96**	**31.32**	0.00
		**6AS-GL**	227.36	3.99	95.73	**422.82**	**139.61**	**47.87**	**5.98**
		8AS-RZ	21.31	0.90	185.74	**506.47**	**153.74**	**64.57**	0.00
		8AS-GL	185.04	0.00	102.36	**263.78**	**141.73**	**39.37**	0.00
		9AS-RZ	21.19	0.95	137.01	**472.61**	**120.80**	**36.62**	0.00
		10AS-RZ	20.72	0.73	133.04	**402.19**	**141.96**	**32.46**	0.00
		11AS-RZ	20.99	0.99	120.78	**255.19**	**119.23**	**27.75**	**17.56**
		12AS-RZ	21.08	0.75	118.71	**394.47**	**129.82**	**29.99**	0.01
		13AS-RZ	24.55	0.91	122.09	**383.50**	**137.10**	**32.13**	2.18
		13AS-AL1	169.76	0.00	71.06	**307.94**	**122.38**	**35.53**	0.00
		**13AS-AL2**	231.17	0.00	95.66	**450.38**	**155.44**	**47.83**	**5.98**
		14AS-AL	25.77	0.59	98.83	**345.08**	**101.55**	**29.94**	0.00
		15AS-RZ	166.14	0.00	94.94	**486.55**	**130.54**	**51.42**	0.00
		15AS-AL1	209.40	0.00	102.73	**659.82**	**134.33**	**51.36**	1.98
		15AS-AL2	39.92	1.42	53.56	**350.33**	**117.92**	**42.36**	0.01
		*x̄*	*85.33*	*0.78*	*105.80*	*384.33*	*130.03*	*37.23*	*2.09*
		SD	*19.99*	*0.00*	*27.94*	*180.55*	*20.03*	*10.79*	*4.24*
		Median	*28.07*	*0.76*	*98.83*	*394.47*	*131.50*	*32.46*	*0.00*
		Min	*81.64*	*0.88*	*36.31*	*108.34*	*91.82*	*20.27*	*0.00*
		Max	*231.17*	*3.99*	*185.74*	*659.82*	*173.96*	*64.57*	*17.56*
	RS	7RS-GL	14.78	0.42	121.20	**255.34**	**141.24**	**27.49**	0.00
20–30	AS	1AS-RZ	35.45	1.16	72.18	**344.69**	**132.29**	**26.03**	0.00
		1AS-GL	147.47	0.00	11.96	**211.24**	**127.54**	**27.90**	**21.92**
		3AS-RZ	25.57	0.93	100.25	77.54	**105.36**	**25.55**	0.01
		5AS-RZ	23.50	0.73	81.42	**389.63**	**120.94**	**28.90**	0.01
		6AS-RZ	20.38	0.70	153.87	63.01	**153.90**	**25.76**	**0.74**
		6AS-GL	176.40	0.00	117.60	**305.76**	**148.96**	**43.12**	**5.88**
		8AS-RZ	19.77	0.82	172.69	**354.73**	**161.65**	**40.34**	0.00
		8AS-GL	202.57	0.00	120.76	**284.38**	**140.24**	**38.96**	**1.95**
		9AS-RZ	23.61	1.33	158.97	**285.26**	**124.12**	**29.77**	0.00
		10AS-RZ	22.55	0.77	119.89	**311.30**	**127.64**	**32.91**	**0.64**
		11AS-RZ	25.09	0.72	125.27	**350.06**	**141.13**	**29.58**	0.00
		12AS-RZ	22.34	0.91	133.83	**388.27**	**113.40**	**22.43**	**2.61**
		13AS-RZ	26.61	0.69	148.11	**432.47**	**123.81**	**33.40**	**4.14**
		13AS-AL1	111.55	0.00	43.82	**278.88**	91.63	**31.87**	**5.98**
		13AS-AL2	119.24	0.00	59.62	**357.71**	**119.24**	**35.77**	**1.99**
		14AS-AL	28.12	0.69	114.75	**387.94**	**104.64**	**27.09**	0.00
		15AS-RZ	195.92	0.00	97.96	**536.83**	**129.31**	**54.86**	0.00
		15AS-AL1	164.00	0.00	108.00	**480.00**	**136.00**	**48.00**	**2.00**
		15AS-AL2	45.79	1.54	67.21	**381.00**	**112.13**	**40.34**	**2.45**
		*x̄*	*75.58*	*0.58*	*105.69*	*327.41*	*127.05*	*33.82*	*2.65*
		*SD*	*19.77*	*0.00*	*11.96*	*63.01*	*17.22*	*8.31*	*4.92*
		*Median*	*28.12*	*0.70*	*114.75*	*350.06*	*127.54*	*31.87*	*0.74*
		*Min*	*67.43*	*0.49*	*40.59*	*114.09*	*91.63*	*22.43*	*0.00*
		*Max*	*202.57*	*1.54*	*172.69*	*536.83*	*161.65*	*54.86*	*21.92*
	RS	7RS-GL	**153.85**	0.00	67.06	**205.13**	**149.90**	**39.45**	0.00
		*SGV*		***7–10***		***100–600***	***100–220***	***5–1000***	***1–20***

*AS* alluvial site, *CS* contaminated site, *RS* reference site, *RZ* riparian zone, *AL* arable land, *GL* grassland, *PEB* probable environmental burden, *CEB* confirmed environmental burden, *SGV* soil guideline value according to Reimann et al.^18^.

Normal letter—below soil guideline value; bold letter—above soil guideline value.

Statistical values are in italics.

**Table 4:** Contamination factor (CF) and pollution load index (PLI) for contaminated and alluvial sites in the depth of 0–10 cm.

Site	Locality	Alkali metal	Alkaline earth metals		Transition metal	Metalloid	PLI
		Li	Sr	Ba	V	B	
CS	2CS-PEB	1.4	0.5	0.1	0.8	0.8	0.5
	4CS-CEB	2.6	1.0	0.4	1.0	1.0	1.0
	*x̄*	*2.0*	*0.7*	*0.3*	*0.9*	*0.9*	*0.8*
	SD	*0.6*	*0.2*	*0.2*	*0.1*	*0.1*	*0.3*
AS	1AS-RZ	2.4	0.7	1.4	0.9	0.9	1.2
	1AS-GL	8.9	0.2	0.9	0.7	1.2	1.1
	3AS-RZ	1.4	0.7	0.7	0.7	0.7	0.8
	5AS-RZ	1.9	0.8	1.6	0.9	1.1	1.2
	6AS-RZ	1.5	1.5	1.6	1.2	1.1	1.4
	6AS-GL	15.4	0.8	1.7	1.0	1.7	2.0
	8AS-RZ	1.4	1.5	2.0	1.1	2.3	1.6
	8AS-GL	12.5	0.8	1.0	1.0	1.4	1.7
	9AS-RZ	1.4	1.1	1.9	0.9	1.3	1.3
	10AS-RZ	1.4	1.1	1.6	1.0	1.2	1.2
	11AS-RZ	1.4	1.0	1.0	0.8	1.0	1.0
	12AS-RZ	1.4	1.0	1.5	0.9	1.1	1.2
	13AS-RZ	1.7	1.0	1.5	1.0	1.2	1.2
	13AS-AL1	11.5	0.6	1.2	0.9	1.3	1.6
	13AS-AL2	15.6	0.8	1.8	1.1	1.7	2.1
	14AS-AL	1.7	0.8	1.4	0.7	1.1	1.1
	15AS-RZ	11.2	0.8	1.9	0.9	1.9	2.0
	15AS-AL1	14.2	0.8	2.6	1.0	1.9	2.2
	15AS-AL2	2.7	0.4	1.4	0.8	1.5	1.2
	*x̄*	*5.8*	*0.9*	*1.5*	*0.9*	*1.4*	*1.4*
	SD	*5.5*	*0.3*	*0.4*	*0.1*	*0.4*	*0.4*
	Min	*1.4*	*0.2*	*0.7*	*0.7*	*0.7*	*0.8*
	Max	*15.6*	*1.5*	*2.6*	*1.2*	*2.3*	*2.2*

*AS* alluvial site, *CS* contaminated site, *RS* reference site, *RZ* riparian zone, *AL* arable land, *GL* grassland, *PEB* probable environmental burden, *CEB* confirmed environmental burden.

The CF < 1 means low contamination, 1–3 is moderate contamination, 3–6 is considerable contamination, and CF > 6 means very high contamination. The PLI > 1 indicates pollution.

Statistical values are in italics.

The potential health risk was assessed by considering oral topsoil ingestion for 7 EECs (Table 5). We discovered possible serious potential health risks only in children, a vulnerable population. The two adult groups are not under the potential health risk by EECs. In the two adult groups, the mean value of HQ for individual EECs ranged from 0.000 to 0.043 for males, and from 0.000 to 0.050 for females. The resultant mean HI values for female persons were 0.040 at CS localities and 0.080 at AS localities. The mean HI values for male persons were 0.034 at CS localities and 0.068 at AS localities. The mean value of HQ for individual EECs ranged from 0.002 to 0.545 for children. The resultant mean HI value for children was 0.435 at CS localities and 0.868 at AS localities. In children, the mean HQ (0.545) at AS localities was found high for Li. Lithium was followed by V (0.238), and Ba (0.070); all the rest of the EECs were much lower and ranged from 0.002 (Sr, B) to 0.005 (Be, Se). The risk for children indicated by HQ for Li was significant at 5 of 15 AS localities where HQ was found high (ranging from 1.085 to 1.478). Two of these 5 localities are used as grasslands, two as arable land and one is a riparian zone.

**Table 5:** Hazard quotient (HQ) and hazard index (HI) for contaminated and alluvial sites in the depth of 0–10 cm.

Group	Site	Locality	Alkali metal	Alkaline earth metals			Transition metal	Metalloid	Non-metal	HI
			Li	Be	Sr	Ba	V	B	Se	
Children	CS	2CS-PEB	0.133	0.006	0.001	0.005	0.204	0.001	0.0	0.351
		4CS-CEB	0.242	0.008	0.002	0.020	0.246	0.002	0.0	0.520
		*x̄*	*0.187*	*0.007*	*0.002*	*0.012*	*0.225*	*0.002*	*0.0*	*0.435*
		SD	*0.054*	*0.001*	*0.001*	*0.008*	*0.021*	*0.000*	*0.0*	*0.084*
	AS	1AS-RZ	0.223	0.006	0.002	0.067	0.244	0.002	0.0	0.544
		1AS-GL	0.842	0.000	0.001	0.042	0.168	0.002	0.015	1.069
		3AS-RZ	0.128	0.005	0.002	0.033	0.170	0.001	0.0	0.339
		5AS-RZ	0.179	0.006	0.002	0.075	0.240	0.002	0.0	0.504
		6AS-RZ	0.142	0.006	0.004	0.076	0.318	0.002	0.0	0.547
		6AS-GL	1.453	0.025	0.002	0.077	0.255	0.003	0.015	1.832
		8AS-RZ	0.136	0.006	0.004	0.093	0.281	0.004	0.0	0.523
		8AS-GL	1.183	0.000	0.002	0.048	0.259	0.003	0.0	1.495
		9AS-RZ	0.135	0.006	0.003	0.086	0.221	0.002	0.0	0.454
		10AS-RZ	0.132	0.005	0.003	0.073	0.259	0.002	0.0	0.475
		11AS-RZ	0.134	0.006	0.003	0.047	0.218	0.002	0.045	0.454
		12AS-RZ	0.135	0.005	0.003	0.072	0.237	0.002	0.0	0.453
		13AS-RZ	0.157	0.006	0.003	0.070	0.250	0.002	0.006	0.493
		13AS-AL1	1.085	0.000	0.002	0.056	0.224	0.002	0.0	1.369
		13AS-AL2	1.478	0.000	0.002	0.082	0.284	0.003	0.015	1.864
		14AS-AL	0.165	0.004	0.002	0.063	0.185	0.002	0.0	0.421
		15AS-RZ	1.062	0.000	0.002	0.089	0.238	0.003	0.0	1.395
		15AS-AL1	1.339	0.000	0.002	0.121	0.245	0.003	0.005	1.715
		15AS-AL2	0.255	0.009	0.001	0.064	0.215	0.003	0.0	0.547
		*x̄*	*0.545*	*0.005*	*0.002*	*0.070*	*0.238*	*0.002*	*0.005*	*0.868*
		SD	*0.522*	*0.006*	*0.001*	*0.020*	*0.037*	*0.001*	*0.011*	*0.536*
		Min	*0.128*	*0.000*	*0.001*	*0.033*	*0.168*	*0.001*	*0.0*	*0.339*
		Max	*1.478*	*0.025*	*0.004*	*0.121*	*0.318*	*0.004*	*0.045*	*1.864*
Adult females	CS	2CS-PEB	0.012	0.001	0.0	0.000	0.019	0.0	0.0	0.032
		4CS-CEB	0.022	0.001	0.0	0.002	0.023	0.0	0.0	0.048
		*x̄*	*0.017*	*0.001*	*0.0*	*0.001*	*0.021*	*0.0*	*0.0*	*0.040*
		SD	*0.005*	*0.000*	*0.0*	*0.001*	*0.002*	*0.0*	*0.0*	*0.008*
	AS	1AS-RZ	0.021	0.001	0.0	0.006	0.023	0.0	0.0	0.050
		1AS-GL	0.078	0.0	0.0	0.004	0.015	0.0	0.001	0.099
		3AS-RZ	0.012	0.0	0.0	0.003	0.016	0.0	0.0	0.031
		5AS-RZ	0.017	0.001	0.0	0.007	0.022	0.0	0.0	0.047
		6AS-RZ	0.013	0.001	0.0	0.007	0.029	0.0	0.0	0.051
		6AS-GL	0.134	0.002	0.0	0.007	0.024	0.0	0.001	0.169
		8AS-RZ	0.013	0.001	0.0	0.009	0.026	0.0	0.0	0.048
		8AS-GL	0.109	0.0	0.0	0.004	0.024	0.0	0.0	0.138
		9AS-RZ	0.013	0.001	0.0	0.008	0.020	0.0	0.0	0.042
		10AS-RZ	0.012	0.0	0.0	0.007	0.024	0.0	0.0	0.044
		11AS-RZ	0.012	0.001	0.0	0.004	0.020	0.0	0.004	0.042
		12AS-RZ	0.012	0.0	0.0	0.007	0.022	0.0	0.0	0.042
		13AS-RZ	0.014	0.001	0.0	0.006	0.023	0.0	0.001	0.046
		13AS-AL1	0.100	0.0	0.0	0.005	0.021	0.0	0.0	0.126
		13AS-AL2	0.136	0.0r	0.0	0.008	0.026	0.0	0.001	0.172
		14AS-AL	0.015	0.0	0.0	0.006	0.017	0.0	0.0	0.039
		15AS-RZ	0.098	0.0	0.0	0.008	0.022	0.0	0.0	0.129
		15AS-AL1	0.124	0.0	0.0	0.011	0.023	0.0	0.0	0.158
		15AS-AL2	0.024	0.001	0.0	0.006	0.020	0.0	0.0	0.051
		*x̄*	*0.050*	*0.0*	*0.0*	*0.006*	*0.022*	*0.0*	*0.0*	*0.080*
		SD	*0.048*	*0.001*	*0.0*	*0.002*	*0.003*	*0.0*	*0.001*	*0.049*
		Min	*0.012*	*0.0*	*0.0*	*0.003*	*0.015*	*0.0*	*0.0*	*0.031*
		Max	*0.136*	*0.002*	*0.0*	*0.011*	*0.029*	*0.0*	*0.004*	*0.172*
Adult males	CS	2CS-PEB	0.010	0.000	0.0	0.000	0.016	0.0	0.0	0.028
		4CS-CEB	0.019	0.001	0.0	0.002	0.019	0.0	0.0	0.041
		*x̄*	*0.015*	*0.001*	*0.0*	*0.001*	*0.018*	*0.0*	*0.0*	*0.034*
		*SD*	*0.004*	*0.0*	*0.0*	*0.001*	*0.002*	*0.0*	*0.0*	*0.007*
	AS	1AS-RZ	0.018	0.001	0.0	0.005	0.019	0.0	0.0	0.043
		1AS-GL	0.066	0.0	0.0	0.003	0.013	0.0	0.001	0.084
		3AS-RZ	0.010	0.0	0.0	0.003	0.013	0.0	0.0	0.027
		5AS-RZ	0.014	0.0	0.0	0.006	0.019	0.0	0.0	0.040
		6AS-RZ	0.011	0.0	0.0	0.006	0.025	0.0	0.0	0.043
		6AS-GL	0.115	0.002	0.0	0.006	0.020	0.0	0.001	0.144
		8AS-RZ	0.011	0.0	0.0	0.007	0.022	0.0	0.0	0.041
		8AS-GL	0.093	0.0	0.0	0.004	0.020	0.0	0.0	0.118
		9AS-RZ	0.011	0.0	0.0	0.007	0.017	0.0	0.0	0.036
		10AS-RZ	0.010	0.0	0.0	0.006	0.020	0.0	0.0	0.037
		11AS-RZ	0.011	0.0	0.0	0.004	0.017	0.0	0.004	0.036
		12AS-RZ	0.011	0.0	0.0	0.006	0.019	0.0	0.0	0.036
		13AS-RZ	0.012	0.0	0.0	0.006	0.020	0.0	0.0	0.039
		13AS-AL1	0.085	0.0	0.0	0.004	0.018	0.0	0.0	0.108
		13AS-AL2	0.116	0.0	0.0	0.006	0.022	0.0	0.001	0.147
		14AS-AL	0.013	0.0	0.0	0.005	0.015	0.0	0.0	0.033
		15AS-RZ	0.084	0.0	0.0	0.007	0.019	0.0	0.0	0.110
		15AS-AL1	0.105	0.0	0.0	0.009	0.019	0.0	0.0	0.135
		15AS-AL2	0.020	0.001	0.0	0.005	0.017	0.0	0.0	0.043
		*x̄*	*0.043*	*0.0*	*0.0*	*0.003*	*0.019*	*0.0*	*0.0*	*0.068*
		SD	*0.041*	*0.0*	*0.0*	*0.002*	*0.003*	*0.0*	*0.001*	*0.042*
		Min	*0.010*	*0.0*	*0.0*	*0.003*	*0.013*	*0.0*	*0.0*	*0.027*
		Max	*0.116*	*0.002*	*0.0*	*0.009*	*0.025*	*0.0*	*0.004*	*0.147*

*AS* alluvial site, *CS* contaminated site, *RS* reference site, *RZ* riparian zone, *AL* arable land, *GL* grassland, *PEB* probable environmental burden, *CEB* confirmed environmental burden.

The HQ and HI > 1 indicate high probability of the occurrence of adverse health effects.

Statistical values are in italics.

### EEC fractionation

**Figure 2:**
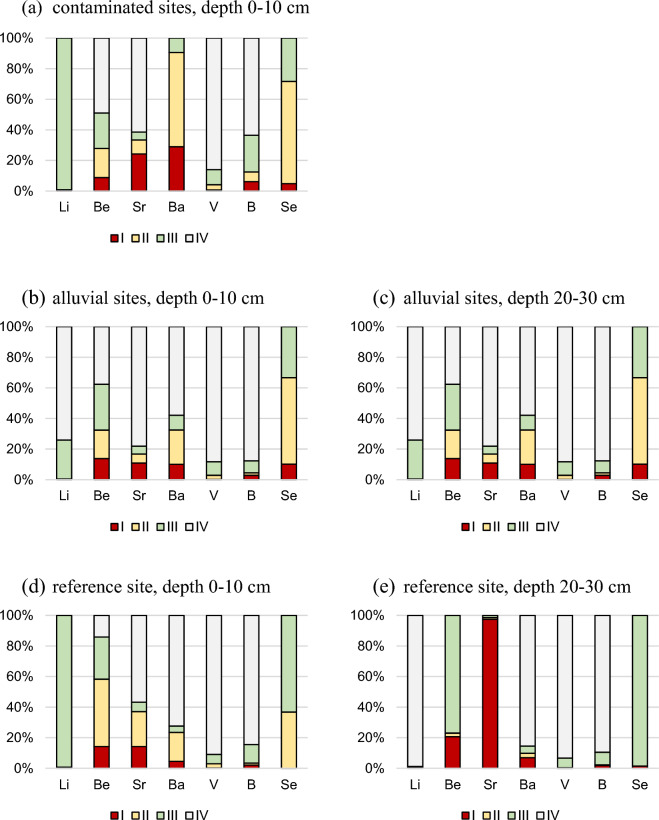
Emerging environmental contaminants distribution in 4 fractions in the depth of 0–10 and 20–30 cm in contaminated, alluvial, and reference sites. I—exchangeable fraction, II—reducible fraction, III—oxidisable fraction, IV—residual fraction.

The content of seven EECs leached in the sequential extraction procedure in the CS, AS, and RS localities and in both depths is summarized in Fig. 2a–e. We recorded different distribution patterns among all four fractions between different elements and related chemical groups of EECs. Between the two different layers among the AS and RS sites, more similar patterns occurred at the AS sites.

V and B showed the highest percentage in the residual fraction (IV) in all CS, AS, and RS sties, and in both depths. This fraction may be considered as biologically unavailable. On the other hand, among all the elements observed, only one element, Sr, showed the highest percentage in the first extractable fraction, which was considered the most mobile in the RS site at the second depth. The third (oxidisable) fraction occurred in the highest percentage in the cases of Li (at CS and in the first depth at RS), Ba (in the first layer at AS and in the second layer at RS), and Se (in the first and second depths at RS). Be appeared in three different fractions (II, III, IV) in the highest percentage at different sites, followed by Li, Sr, Ba, and Se in two different fractions in the highest percentage at different sites. V and B occurred in only one (IV) fraction of the highest percentage at all sites.

Alkali earth metals (Be, Sr, and Ba) reflected the most equable distribution between fractions together with B and Se in some cases. The distribution of the alkali earth metals in the different fractions mostly associated with the first two most mobile fractions (I and II) indicated that the mobility declined on the order of Ba, Sr, and Be, at CS sites; Ba, Be, and Sr at AS sites; Be, Sr, and Ba at the RS site in the first depth and Sr, Be, and Ba in the second depth, respectively.

At AS localities in the first depth, the average content of Ba represented 10.0% in exchangeable, 22.5% in reducible, and 9.6% in oxidisable fraction; the content of V represented 0.2% in exchangeable, 2.7% in reducible, and 8.8% in oxidisable fraction; the content of B represented 2.9% in exchangeable, 1.6% in reducible, and 7.8% in oxidisable fraction; and the content of Se represented the same.

### EEC fractions relationships with soil properties, ecosystem types, and soil depth

The correlation coefficient matrix for the EEC fractions with the chemical and physical parameters of the soil is shown in Table 6. The most numerous significant correlations with chemical properties occurred in the case of B (17), while the least occurred in the case of Li (6). The most numerous significant correlations with physical properties occurred in the case of Sr (12) while no correlations occurred in the case of Se (0). Among chemical properties, we most often observed correlations with pH/KCl (18 out of 35), followed by SOC (14 out of 35), Mn_o_ (13 out of 35), pH/H_2_O (10 out of 35), HA/FA (10 out of 35), Fe_o_ (5 out of 35), Eh (4 out of 35) and Al_o_ (3 out of 35). Only in one case of boron, there was correlations between all four fractions, including the total content and the oxalate-extractable Mn_o_. Among physical properties, we most typically observed correlations with the silt and sand content (9 out of 35), followed by the clay content and salinity (7 out of 35).

**Table 6:** Pearson’s correlation coefficient matrix of EECs in various fractions and chemical and physical properties of the soil (n = 42).

		pH/H_2_O	pH/KCl	Eh	Al_o_	Fe_o_	Mn_o_	SOC	HA/FA	Content of			SAL	SM
										Clay	Silt	Sand		
Li	I	N.C	N.C	N.C	N.C	N.C	N.C	N.C	N.C	N.C	N.C	N.C	N.C	N.C
	II	N.C	0.326*	N.C	N.C	N.C	N.C	0.306*	N.C	N.C	N.C	N.C	N.C	N.C
	III	N.C	0.346**	N.C	N.C	N.C	N.C	0.427**	N.C	N.C	N.C	N.C	N.C	N.C
	IV	N.C	N.C	N.C	N.C	N.C	N.C	− 0.417**	N.C	N.C	0.426**	− 0.352*	N.C	N.C
	T	N.C	N.C	N.C	N.C	N.C	N.C	− 0.368*	N.C	N.C	0.429**	− 0.353*	N.C	N.C
Be	I	N.C	N.C	N.C	N.C	N.C	N.C	N.C	N.C	N.C	0.323*	− 0.322*	N.C	N.C
	II	0.408**	0.355*	N.C	N.C	N.C	N.C	0.479**	N.C	0.401**	N.C	N.C	N.C	N.C
	III	0.484**	0.490*	N.C	N.C	N.C	0.673**	N.C	0.366*	0.323*	N.C	N.C	N.C	N.C
	IV	N.C	N.C	N.C	N.C	N.C	N.C	N.C	N.C	N.C	N.C	N.C	N.C	N.C
	T	N.C	N.C	N.C	N.C	N.C	N.C	0.327*	N.C	N.C	N.C	N.C	N.C	N.C
Sr	I	N.C	N.C	N.C	0.490**	N.C	N.C	N.C	N.C	N.C	N.C	N.C	N.C	N.C
	II	N.C	N.C	N.C	0.375*	N.C	N.C	0.603**	N.C	0.454**	N.C	N.C	N.C	N.C
	III	N.C	N.C	N.C	N.C	N.C	N.C	0.371*	− 0.551**	N.C	− 0.715**	0.656**	N.C	N.C
	IV	N.C	N.C	N.C	− 0.500**	N.C	− 0.320*	N.C	− 0.330*	− 0.486**	0.418**	0.503**	− 0.392*	− 0.348*
	T	− 0.386*	− 0.377*	N.C	N.C	N.C	− 0.499**	N.C	− 0.535**	− 0.403**	0.611**	0.623**	− 0.316*	N.C
Ba	I	N.C	N.C	N.C	N.C	N.C	N.C	N.C	N.C	N.C	N.C	N.C	N.C	N.C
	II	N.C	N.C	N.C	N.C	0.305*	N.C	N.C	N.C	N.C	− 0.546**	0.451**	N.C	N.C
	III	− 0.361*	− 0.331*	N.C	N.C	N.C	N.C	N.C	− 0.522**	N.C	− 0.599**	0.573**	− 0.450**	N.C
	IV	N.C	− 0.309*	N.C	N.C	− 0.502**	N.C	N.C	N.C	N.C	0.520**	− 0.460**	N.C	N.C
	T	N.C	− 0.321*	N.C	N.C	− 0.450**	− 0.375*	N.C	N.C	N.C	N.C	N.C	− 0.307*	N.C
V	I	0.678**	0.767**	N.C	N.C	N.C	0.464**	0.456**	0.356*	N.C	N.C	N.C	N.C	N.C
	II	N.C	N.C	N.C	N.C	N.C	N.C	N.C	N.C	0.467**	N.C	N.C	N.C	N.C
	III	N.C	N.C	N.C	N.C	N.C	N.C	0.530**	N.C	N.C	N.C	N.C	N.C	N.C
	IV	− 0.386*	− 0.405**	0.382*	N.C	N.C	− 0.316*	N.C	− 0.309*	N.C	N.C	N.C	− 0.384*	N.C
	T	− 0.367*	− 0.387*	0.385*	N.C	N.C	− 0.346*	N.C	− 0.331*	N.C	N.C	N.C	− 0.395**	N.C
B	I	0.762**	0.780**	− 0.457**	N.C	N.C	0.434**	0.357*	0.425**	0.362*	N.C	N.C	0.478**	N.C
	II	0.438**	0.508**	− 0.388*	N.C	0.322*	0.411**	0.480**	N.C	N.C	N.C	N.C	N.C	N.C
	III	N.C	0.313*	N.C	N.C	N.C	0.453**	0.333*	N.C	N.C	N.C	N.C	N.C	N.C
	IV	N.C	N.C	N.C	N.C	N.C	− 0.541**	N.C	N.C	N.C	N.C	N.C	N.C	N.C
	T	N.C	N.C	N.C	N.C	N.C	− 0.465**	N.C	N.C	N.C	N.C	N.C	N.C	N.C
Se	I	N.C	0.337*	N.C	N.C	N.C	N.C	N.C	N.C	N.C	N.C	N.C	N.C	N.C
	II	0.561**	0.645**	N.C	N.C	0.381*	0.748**	N.C	0.391*	N.C	N.C	N.C	N.C	N.C
	III	N.C	0.338*	N.C	N.C	N.C	N.C	0.362*	N.C	N.C	N.C	N.C	N.C	N.C
	IV	N.C	− 0.341*	N.C	N.C	N.C	N.C	N.C	N.C	N.C	N.C	N.C	N.C	N.C
	T	N.C	N.C	N.C	N.C	N.C	N.C	N.C	N.C	N.C	N.C	N.C	N.C	N.C

*I* exchangeable fraction, *II* reducible fraction, *III* oxidisable fraction, *IV* residual fraction, *T* total content, *Eh* redox potential, *SOC* soil organic carbon, *HA/FA* ratio of humic acid carbon to fulvic acid carbon, _*o*_ extracted by oxalate, *SAL* salinity, *SM* soil moisture; *r-values shown in bold are significant at P < 0.05; **r-values shown in bold are significant at P < 0.01; *N.C.* no correlation.

**Figure 3:**
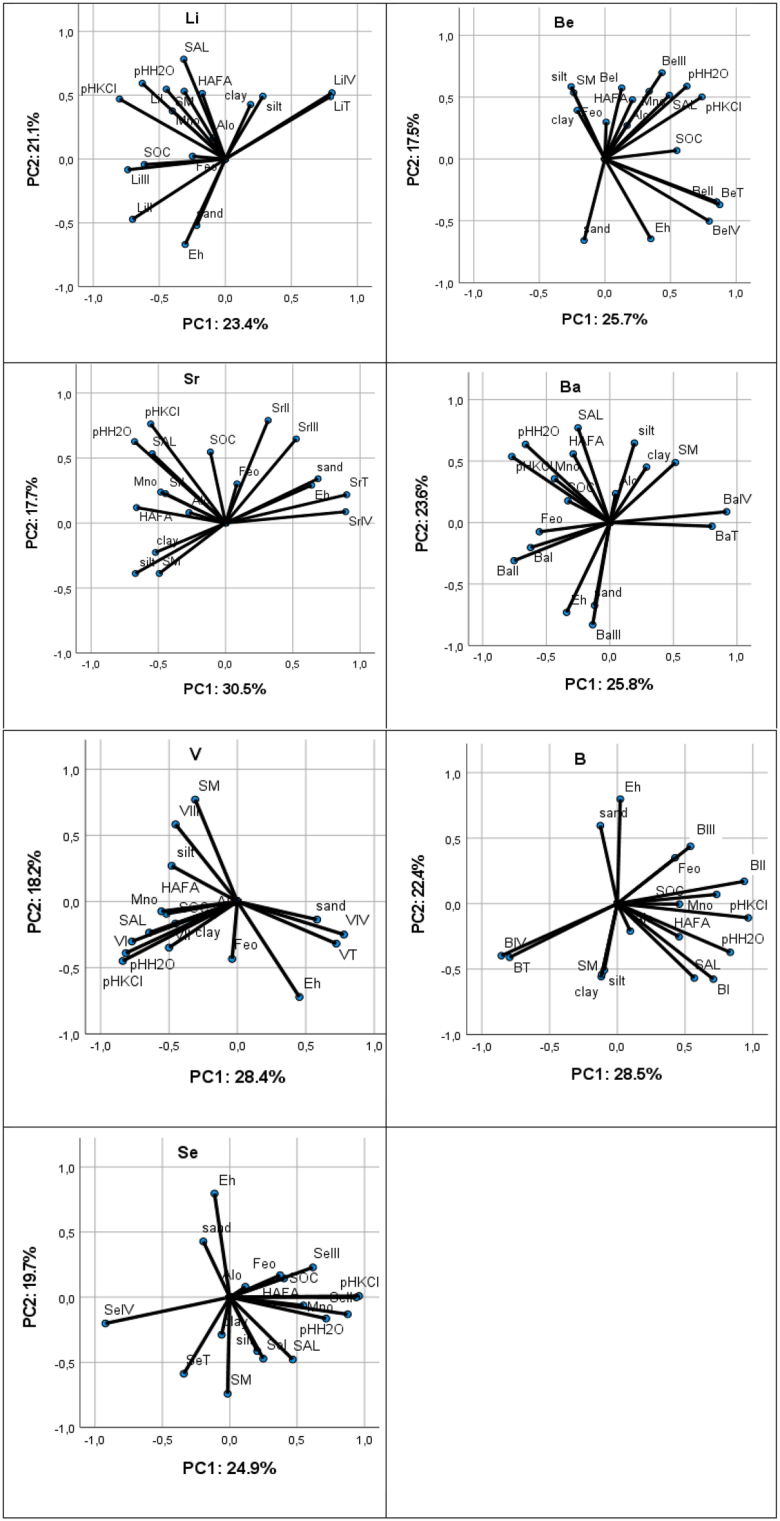
PCA relationships between EECs and soil properties. I—exchangeable fraction, II—reducible fraction, III—oxidisable fraction, IV—residual fraction, T—total content.

The PCA showed that the first two factors can explain more than 43% of the total variance for all EECs, from 43.2% in the case of Be to 50.9 in the case of B (Fig. 3). Salinity and pH/H_2_O were positively and Eh negatively correlated with PC2 for Li and Ba. pH/KCl was clearly positively correlated with PC1 for Be. pH was positively correlated with PC2 and silt was negatively correlated with PC1 for Sr. Soil moisture was positively correlated with PC2 and pH negatively correlated with PC1 for V. pH was clearly strongly positively correlated with PC1 and Eh with PC2 for B and Se.

To evaluate the significance of the difference between mean values of each EEC fraction, one-way ANOVA was used. The independent variables were two different ecosystem types (riparian zone and agricultural ecosystem represented by arable land and grassland) and soil depths (0–10 and 20–30 cm), while the dependent variables were the EEC fractions. The result of ANOVA test is presented in Table 7. The statistical analysis showed significant differences between the ecosystem types and all Ba fractions, including the total content. Significant differences appeared between the different ecosystem types for Li and Sr in fractions III, IV and total content, for B and Se in fractions II and III, and for V in fraction I. The ANOVA showed significant differences between the soil depth and the EEC fractions only in the case of Li (I).

**Table 7:** Results of ANOVA for the EEC fractions by ecosystem type and soil depth (n = 40).

		Ecosystem type		Soil depth	
		*F*-value	*P*-value	*F*-value	*P*-value
I	Li	1.235	0.312	2.760	0.106
	Be	0.166	0.919	0.179	0.675
	Sr	1.494	0.233	2.601	0.116
	Ba	9.971	**˂ 0.001**	0.039	0.844
	V	14.404	**˂ 0.001**	3.303	0.078
	B	0.210	0.889	0.053	0.819
	Se	0.115	0.950	0.378	0.543
II	Li	0.224	0.879	3.011	0.091
	Be	2.791	0.055	1.634	0.210
	Sr	0.870	0.466	1.421	0.241
	Ba	12.523	**˂ 0.001**	0.052	0.821
	V	2.167	0.109	0.307	0.583
	B	8.097	**˂0.001**	0.044	0.836
	Se	2.485	0.077	0.011	0.919
III	Li	7.872	**˂ 0.001**	15.025	**˂ 0.001**
	Be	0.076	0.972	0.199	0.659
	Sr	6.402	**0.001**	0.665	0.420
	Ba	10.742	**˂ 0.001**	0.131	0.720
	V	1.688	0.187	1.727	0.197
	B	8.711	**˂ 0.001**	2.872	0.099
	Se	5.039	**0.005**	0.432	0.515
IV	Li	9.777	**˂ 0.001**	0.355	0.555
	Be	0.815	0.494	2.300	0.138
	Sr	3.461	**0.027**	1.315	0.259
	Ba	6.338	**0.002**	0.784	0.382
	V	0.916	0.443	0.029	0.866
	B	2.532	0.073	0.241	0.626
	Se	3.321	**0.031**	0.873	0.356
T	Li	9.530	**˂ 0.001**	0.015	0.904
	Be	0.866	0.468	2.224	0.145
	Sr	4.961	**0.006**	0.129	0.722
	Ba	6.683	**0.001**	1.784	0.190
	V	1.409	0.256	0.033	0.858
	B	1.645	0.197	0.467	0.499
	Se	1.661	0.193	0.877	0.355

P-values highlighted in bold are highly statistically significant (P < 0.001) and statistically significant (P < 0.05).

**Figure 4:**
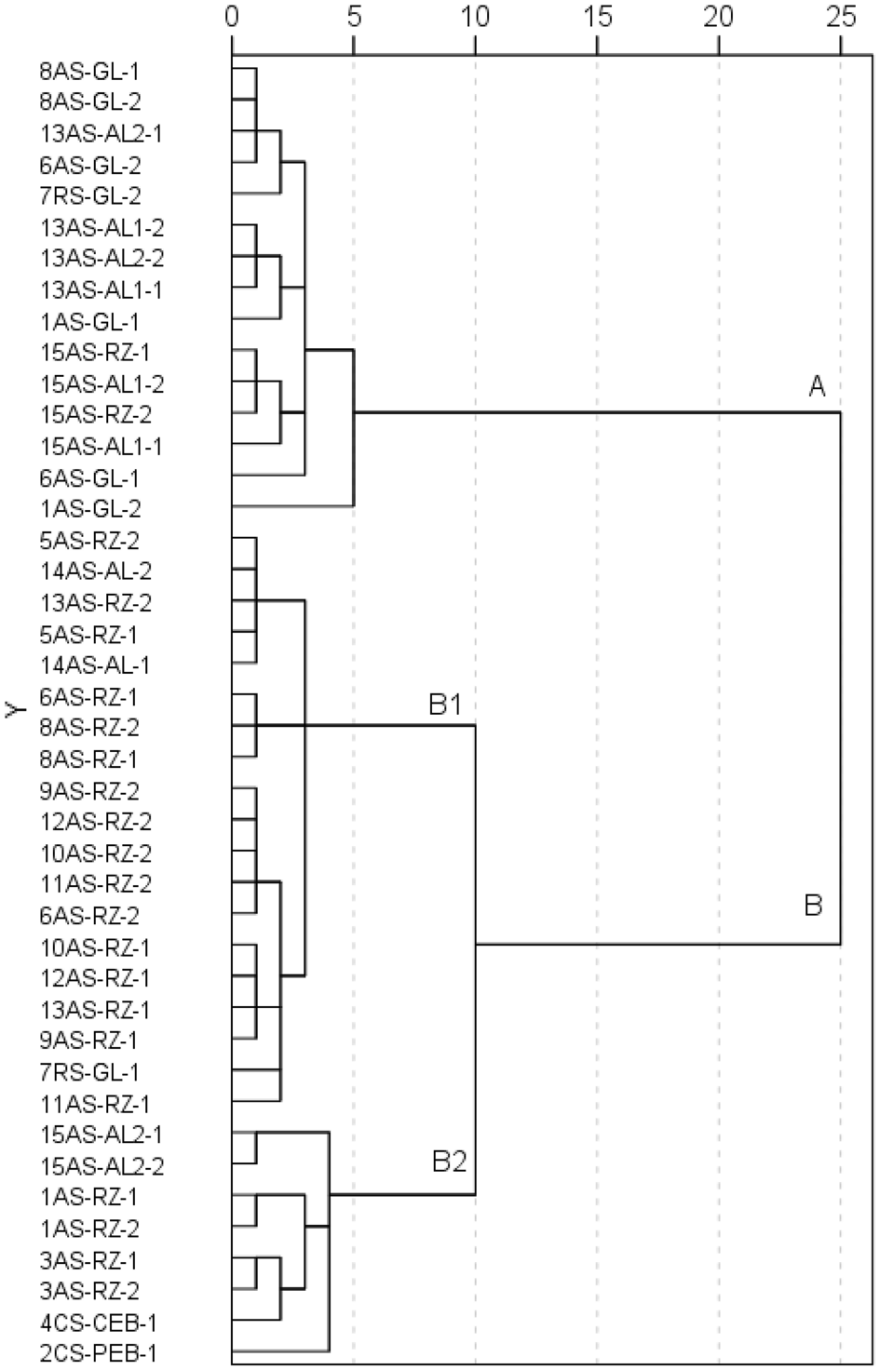
Dendrogram for the hierarchical clustering of sites (Ward’s method).

The HCA (Fig. 4) has highlighted two large clusters (A and B). Cluster A contains sites that are primarily used as agricultural land (GL and AL). Two clusters of lower hierarchical order are recognised in B (B1 and B2). Cluster B1 includes predominantly RZ sites (16 out of 19). Cluster B2 includes five sites, two of which are contaminated sites.

## Discussion

Based on the available SGVs, we discovered four EECs (Ba, V, B, Se) contaminating the entire SR floodplain. The SGV value exceeded was not only reported in the case of Be. The SGVs were not available for Li and Sr. According to the calculated contamination indices for five EECs (Li, Sr, Ba, V, and B), Li is the most serious EEC of the SR floodplain, followed by Ba and B. Based on the calculated contamination indices for 5 EECs (Li, Sr, Ba, V, B), mainly Li and fewer Ba, B are the most serious EECs of the SR floodplain. Based on the content of selected EECs in soil and calculated contamination and pollution indices, many AS localities are more contaminated by selected EECs than the confirmed environmental burden (4CS-CEB). In addition, some of them are used intensively for agricultural production. Although the contents of the selected EECs exceeded some SGVs, the contamination indices indicate mainly moderate contamination. Therefore, they are probably not of interest for further industrial use in the sense of soil mining. The demand for new raw materials is growing very quickly, and other raw or natural materials are already being used today for the extraction of CRM. In recent times, many CRMs are seldom mined as the main ore. They are usually coproducts of the principal ore. Therefore, historical mining regions may be of such interest^19^. Many tailings did not meet the requirements of further industrial production and were only piled up as waste^20^. In addition, tailing deposits have become potential contamination source^21^. Thus, they impacted the surrounding environmental compartments. The proof of this is the SR floodplain contaminated by heavy metals as reported by Kanianska et al.^22^ and other pollutants. The HMs together with EECs released during mining processes were transported and spread along the floodplain. Rivers and alluvial soils are affected by discharges from mines and tailings^23^. In addition, polluted aquifers act as long-term pollution source to the surrounding gaining rivers and soils, even after the mining activities were stopped^24^. Despite the considerable scale, there are many such territories with limited data availability^25^. However, they can pose ecological and health risks with respect to the cumulative effect of possible additional contamination from existing and potential new pollution sources. In our study, Li exhibited the highest mean contamination factor with a value of 5.8, followed by Ba with 1.5 and B with 1.4. We also found possible potential health risk for children in the case of Li at 5 of 15 alluvial localities with HQ higher than 1.

Different contaminants with different chemical properties can contribute to these risks and interact with each other under the simultaneous effect of the soil environment and its specific properties. Therefore, research on the most serious EEC behaviour in soil in relation to soil properties is one of the key factors to prevent or decrease ecological and health risks. The BCR SEP method allowed us to study the mobility and availability of EECs. This method is accepted by many researchers dealing with toxic metals in mining waste (e.g., Fernandez-Ondono et al.^26^). Although there are authors who believe that the application of this procedure can lead to ambiguous and misleading interpretation^27^. This method is still being increasingly applied in EEC analyses (e.g. Chen et al.^28^, Mikavica et al.^29^, Xiong et al.^30^).

### Lithium and its behaviour in floodplain contaminated soils

Lithium does not belong to the essential nutrients group. But there is evidence that low Li levels have beneficial effects on living organisms^31^. Lithium possesses unique essential and neurophysiological characteristics in biota and humans^32^. However, at high doses, it can be toxic to humans^33^.

The Li content in the soil is variable from place to place. Upper layers usually contain less Li than the underlying layers^34^, as markedly the case at the RS locality. In the AS localities, the total Li content was mostly higher in the first depth, but at 7 out of 19 localities it was the opposite. The average Li content in European agricultural soils is 11.4 mg/kg, with a predominantly low concentration (median 6.4 mg/kg) in northern Europe and higher values (median 15 mg/kg) in southern Europe^35^. We recorded a total Li content of 14.78 mg/kg in the RS locality and 29.29 mg/kg as a mean value in the CS localities. A significantly higher total Li content was recorded in AS localities used in agriculture with a mean value of 152.52 mg/kg compared to localities in the riparian zone with a mean value of 36.46 mg/kg. Such content can pose an environmental risk. In the CS localities, the average CF (2.0) showed moderate contamination by Li. At AS localities, the average CF (5.8) showed considerable contamination by Li.

We also discovered serious potential health risks for children, a vulnerable population. The mean HQ (0.545) at AS localities was found to be high for Li. The risk for children indicated by HQ for Li was significant at 5 of 15 AS localities where HQ was found high (ranging from 1.085 to 1.478). Lithium toxicity results in abnormalities and dysfunctions in several metabolic pathways, causing serious problems for human health^3^.

The toxicity of EECs is also related to the distribution of their fractions. Li was heterogeneously distributed in fractions among different ecosystem types. Exchangeable and reducible fractions showed up in all land use types and in both layers with the lowest Li content, accounting for ˂ 1% Li, which is in line with the findings of other authors^36^. The residual fraction demonstrated the highest Li content at the AS localities in both depths and at the RS locality in the deeper layer. At the CS and in the first depth at the RS locality, Li demonstrated the highest content in the oxidisable fraction. This was confirmed by correlation analyses, where we found positive correlations between the reducible and oxidizable Li fractions and the SOC, similarly as with pH/KCl. Motowicka-Terelek et al.^37^ also found correlations between soil Li content and SOC, but there are also authors who did not^36,38^. From the physical properties, the fine granulometric fraction (silt) showed a positive correlation with the residual fraction and the total Li content. Kabata-Pendias and Mukherjee^39^ refer to the texture, respectively, the fine clay granulometric fraction as the most important factors controlling the Li status of soil. They consider SOC and pH to be factors of lesser importance.

### Barium and its behaviour in floodplain contaminated soils

Barium is not an essential nutrient for humans. But it probably fulfils biochemical functions important for animals and plants^40^. The content of barium in food does not usually pose a health risk. Its long-term impact on human health is questionable^41^. Barium toxicity is related to the solubility of the compound^42^.

The general threshold and guideline values for Ba have not been developed. Only a few countries in the world have adopted them. The Ba limit for food safety has not yet been established^43^. Furthermore, geographic variation is little known^44^. Anthropogenic releases are primarily associated with industrial activities, including mining, and pesticide application in agriculture^45^. Barium compounds are quickly dissolved and can be widely spread, as was also the case with the SR.

In AS localities, the total Ba content was mostly higher in the first depth, but at 6 out of 19 localities, it was the opposite. The median content of Ba in European agricultural soil is 62 mg/kg, with predominantly low concentrations (median 43 mg/kg) in Northern Europe and higher values (median 74 mg/kg) in Southern Europe^18^. We recorded a total Ba content of 255.34 mg/kg in the RS locality and 66.91 mg/kg as a mean value in the CS localities. A higher total Ba content was recorded in AS localities with a mean value of 384.33 mg/kg (median value of 394.47 mg/kg), ranging from 108.34 to 659.82 mg/kg in the first soil layer. González-Valoys et al.^46^ measured in the mine soils in Panama an average total Ba content of 200.6, and in the surrounding soils 349.7 mg/kg. Norini et al.^43^ measured in the mine soil of a settling pond of the Ag–Pb ore mine in France 1701 mg/kg of total Ba and 27.2 mg/kg of water-soluble and cation exchange Ba fraction. At the AS localities, we measured the mean content of Ba in the I exchangeable fraction of 38.58 in the upper layer, ranging from 10.27 to 79.77 mg/kg.

The oxidisable fraction demonstrated the lowest Ba content in all types of land use and in both layers, accounting for 4.14–10.81% Ba. The Ba is thought to be incapable of forming organic ligands or complexes with fulvic and humic acids^47^. This was also confirmed by correlation analysis, where we did not find correlations between the Ba fractions and the SOC. We recorded only negative correlations between the oxidisable Ba fraction and the HA/FA ratio. In the AS and RS localities, the residual fraction showed the highest content, accounting for 51.7 to 85.44% Ba. Ba was heterogeneously distributed in extractable and reducible fractions between different types of land use and soil layers. The reducible and extractable fractions showed the highest Ba content in CS localities. The extractable fraction demonstrated on average 10.0% Ba at AS localities in the first layer and 11.6% in the second layer. Cappuyns^44^ found low Ba mobility in alluvial soils of the Schelter and Demer rivers in Flanders (˂ 3% of the total content). Ba adsorbs into Fe hydroxides^48^, and we also found a positive correlation between the reducible Ba fraction and Fe_o_. The pH of the soil can influence barium behaviour and is more mobile under acidic conditions. The pH showed negative correlations with the total Ba content, oxidisable fractions, and residual fractions. In contrast to Frohne et al.^49^, we did not find correlations with Eh. Similarly, we did not find correlations with clay particles, as was found by Atun and Bascetin^50^. The reducible and oxidisable Ba fractions were positively correlated with the content of the sand particles. Only the IV Ba fraction was positively correlated with the silt content and negatively correlated with the content of the sand particles.

### Boron and its behaviour in floodplain contaminated soils

Boron at low concentrations is probably an essential element for plants^51^ humans and animals, while it is toxic at high concentrations^52^. But recently, B was assumed to be rather toxic to plant cells at low levels^53^. Boron has an extremely narrow range between deficiency and toxicity^54^.

In AS localities, the total B content was mostly higher in the first depth, but at 6 out of 19 localities it was the opposite. The median content of B in European agricultural soil is 2.42 mg/kg, with a predominantly low concentration (median 1.7 mg/kg) in northern Europe and higher values (median 3 mg/kg) in southern Europe^18^. Near a large boron mine in Turkey, Alan and Kara^55^ measured the total B content in soil from 36.5 to 820.5 mg/kg. We recorded a total B content of 27.49 mg/kg at the RS locality and 25.56 mg/kg as the mean value at the CS localities. In AS localities, the mean value was 37.23 mg/kg (median value 32.46 mg/kg), ranging from 20.27 to 64.57 mg/kg in the first soil layer.

Although the total B content exceeds the soil guideline value in most AS localities, sequential extraction showed the highest percentage of residual fraction (IV) in all CS, AS and RS sties, and at both depths. On the other hand, the percentage of mobile and other fractions was low (2.9% in exchangeable, 1.6% in reducible and 7.8% in oxidisable fraction). Similarly, Alan and Kara^55^ found the highest B content in the residual fraction. Therefore, most of the boron that occurs in the soil is unavailable to plants^56^.

We found the most correlations between the B fractions and the physicochemical properties. However, the Eh was negatively correlated with extractable and reducible fractions of B. The pH, SOC, and Mn_o_ oxides were positively correlated with the extractable, reducible, and oxidisable fractions of B. The extractable B fraction was also positively correlated with HA/FA. Hou et al.^57^ also found positive correlations between readily soluble B and pH and between the oxidisable fraction and SOC. From the physical properties, the extractable fraction correlated positively with the clay content of the soil in the study area.

## Conclusions

We detected some level of EEC contamination, particularly by Li, Ba, and B in the floodplain soils of the SR. This contamination could originate simultaneously with HM contamination, mainly from historical mining. The downstream transport could be a source of EECs as accessories to HMs for distant soils.

Sequential extraction technique was used to assess the environmental risk of EECs. The partitioning of the EECs showed that the EECs are mainly associated with the most stable (biologically unavailable) residual fraction, except for Be and Se. The risk of these two elements is reduced by their low (even zero) total contents in the soil. The exchangeable fraction as the most bioavailable occurred in the first soil layer at the AS localities in the highest percentage for Be (13.8%) followed by Sr (10.9%), Se (10.2%), Ba (10.0%) and B (2.9%). The Li and V showed the lowest percentage in the fraction (less than 0.3%). However, the potentially available fractions represented by the II and III fractions represented a higher percentage in the case of Ba (22.5% in reducible and 9.6% in the oxidizable fraction) and Li (25.5% in the oxidizable fraction).

The soil parameters were selectively correlated with the EEC fractions, and we most often observed correlations with pH/KCl, followed by SOC and manganese hydroxides.

The highest mean value of the contamination factor was calculated for Li (5.8), followed by Ba (1.5) and B (1.4). We also found a possible potential health risk for children in the case of Li (HQ ranged from 0.128 to 1.478). The results underscore the significance of recognizing the interconnections between environmental and human health. Such integrating framework of environmental risk assessment posed by EECs comprising chemical and ecotoxicological lines of evidence can improve decision-making processes and help to manage contaminated sites.

## Data Availability

The datasets used for the current study are available from the corresponding author on request.
